# Supplementary material for: Ethanolic extract of Otostegia fruticosa induces ROS-dependent apoptosis and reduces migration of MDA-MB-231 cells in vitro
Source: PLoS One. 2026 May 26;21(5):e0349314. doi: 10.1371/journal.pone.0349314 (PMC13210379; doi:10.1371/journal.pone.0349314)
Supplement: Attachment — Submitted filename: renamed_3c14b.doc [file pone.0349314.s002.doc]

**Author’s Response to the Editors and referees Comments**

**Journal**	: PLOS one

**Manuscript Title**	: Molecular insights into the apoptotic and metastatic effects of *Otostegia fruticosa* on triple-negative breast cancer cells (MDA-MB-231)

**Manuscript Number.** : PONE-D-25-10876

**Article Type**	: Original Research

Subject	**:** Submission of revised manuscript

Thank you for your email enclosing the reviewers’ comments. We appreciate the time and effort by editor and each of the reviewers have dedicated to providing insightful feedback on ways to strengthen our manuscript. We have carefully gone through the comments and responded to the points raised by the reviewers. A detailed response to each of the reviewer’s comments are given below.

**Author’s Reply to referees Comments:**

**Response to Reviewer #1**

We sincerely thank the reviewer for the valuable, insightful, and encouraging remarks on our work. We are particularly grateful for the reviewer's constructive comments that help us improve the clarity and quality of the manuscript. We have revised the manuscript according to the reviewer’s feed backs and concerns as detailed below.

Comment #1: It would greatly enhance the readability and organization of the manuscript if page numbers were included. This would facilitate easier navigation for reviewers and readers, allowing them to reference specific sections more efficiently

**Response:** Thank you for pointing that out. We've added the page number and corrected the error

Comment #2: The title "Molecular insights of apoptotic/metastasis effects of Otostegia fruticosa Forssk. on triple-negative breast cancer cells (MDA-MB-231)" is generally suitable for a scientific manuscript publication. It is specific, informative, and highlights key aspects of the study. Suggestions for improvement to further refine the title: "Molecular insights into the apoptotic and metastatic effects of Otostegia fruticosa on triple-negative breast cancer cells (MDA-MB-231)."

**Response:** Thank you for the suggestion. We have chosen the title: 'Molecular insights into the apoptotic and metastatic effects of Otostegia fruticosa on triple-negative breast cancer cells (MDA-MB-231)

Comment #3: The abstract does not follow a structured approach, which usually starting with an introduction to the study's focus, followed by methodology, results, and conclusions.

**Response:** Thank you for your feedback. We have restructured the abstract for better clarity.

Comment #4: The abstract jumps directly into the study without providing introduction/background on why Otostegia fruticosa Forssk. are of interest or the significance of the research question. Briefly introduce the research problem or question and its significance in the abstract.

**Response:** Thank you for your comment. We have included the introduction and background information on Otostegia fruticosa Forssk.

Comment #5: While necessary for specificity, technical terms like "MTT, ROS, qRT-PCR, MMP9, OPN, etc.” might be unfamiliar to non-experts. Consider to use the full term the first time they appear in the article if possible.

**Response:** Thank you for pointing that out. We have included the full term as suggested.

Comment #6: The methods mentioned in the abstract must align with those in the full Methodology section.

**Response:** Thank you for your comment. We have revised the abstract based on the methodology section to ensure consistency.

Comment #7: The conclusion part does not clearly emphasize the potential impact of the main findings.

**Response:** Thank you for your feedback. We have revised and corrected the conclusion section.

Comment #8: Change “Otostegia Fruticosa” to “Otostegia fruticosa”. It is a Latin name. The second part, always in lowercase.

**Response:** Thank you for the suggestion. We have adopted the recommended term.

Comment #9: The keywords used were relevant for searchability.

**Response:** Thank you for your feedback. We have revised and improved the keywords.

Comment #10: However, the key studies that form the foundation of the research should be properly cited. For instance, in lines 55–57, the authors state that 'Recent studies have validated its ethnopharmacological uses,' but the references cited are outdated. Three out of five references are 8, 25, and 30 years old. It is advisable to use references from the last 5 to 10 years, especially in rapidly evolving fields like medicine.

**Response:** Thank you for pointing that out. The error has been corrected.

Comment #11: In line 59, the format of the citations at the end of the sentence should be checked to ensure it follows the acceptable format.

**Response:** We've rectified the error, thank you for the suggestion.

Comment #12: The authors have clearly stated the problem statement, which is commendable. However, it would strengthen the manuscript if they could provide a more detailed explanation of the current treatments for triple-negative breast cancer (TNBC) and discuss the limitations and challenges associated with these approaches. This additional context would enhance the reader's understanding of the significance of their research.

**Response:** We have included current treatments for triple-negative breast cancer (TNBC) and discussed their limitations.

Comment #13: In Lines 91–92, under the subtopic “Chemicals used”, the authors mention that the materials (cell culture reagents such as DMEM, antibiotics, and fetal bovine serum) were procured from American suppliers. It would enhance the manuscript's transparency and reproducibility if they could provide specific details about the American suppliers from whom the chemicals and materials were obtained. This information is valuable for readers who may wish to replicate the study.

**Response:** Thank you for your feedback. We have rephrased the sentences for clarity.

Comment #14: In Lines 105, 113, 116, 124, 129, 131, 134, 139, 140, 147, 154, 162, 169, 176, 183, 191, 196, 217, change “Otostegia Fruticosa” to “Otostegia fruticosa”. It is a Latin name. The second part, always in lowercase.

**Response:** We have incorporated the suggestions as per your feedback

Comment #15: Under the subtitle 'Preparation of Otostegia fruiticosa ethanol extract,' the authors provide a general overview but do not include sufficient detail regarding the extraction process using a Soxhlet apparatus. It would be beneficial if they could elaborate on the specific methodology, including the percentage of ethanol used and the duration of the extraction. Providing these details will enhance the clarity and reproducibility of the experimental procedure.

**Response:** We appreciate the reviewer's comment and have added sufficient data accordingly.

Comment #16: Under subsection “Cytotoxicity Evaluation Using MTT Assay”, in Line 112, please provide the full name of MTT before its abbreviation is introduced. This will ensure that all readers, including those unfamiliar with the term, can understand the manuscript clearly when the abbreviation is used later.

**Response:** The full form of MTT (3-(4,5-Dimethylthiazol-2-yl)-2,5-diphenyltetrazolium bromide) has been provided.

Comment #17: In line 116, I recommend that the authors provide detailed information regarding the concentrations of the plant extract used in their experiments. This addition will improve the clarity and scientific rigor of the manuscript.

**Response:** Thank you for pointing that out. We have included the concentration of the plant extract used.

Comment #18: In lines 122 – 127, under subsection “Assessment of apoptotic cells via trypan blue exclusion”, the word “trypan” is typically written with a capital “T” in scientific literature.

**Response:** Thank you for pointing out the error. We have corrected it.

Comment #19: In lines 122–127, the subsection titled “Assessment of apoptotic cells via trypan blue exclusion” raises some concerns. The Trypan Blue Exclusion Method is primarily used to assess cell viability and is not specifically suitable for evaluating apoptotic cells. I would appreciate clarification on how the authors justify the use of this method for assessing apoptosis. Additionally, it may be beneficial to consider using more specific assays designed for apoptosis detection, such as Annexin V staining or caspase activity assays.

**Response:** Thank you for your comment. The Trypan blue exclusion assay is primarily used to assess cell viability. However, apoptotic cells were identified microscopically due to changes in membrane integrity. We quantified apoptotic cells and presented the results graphically.

Comment #20: In line 130, the unit for the concentrations of O. fruticosa extract should be expressed more appropriately. Clear and consistent units are essential for ensuring accurate interpretation of the data. I recommend revising this line to specify the unit used for concentration, such as μg/mL, to enhance clarity.

**Response:** Thank you for the feedback. We have revised the concentration unit accordingly.

Comment #21: In lines 129-130, under the subsection 'Cell viability analysis,' you mention testing a range of concentrations (0, 23, and 46 μg) at multiple time points. I would appreciate clarification on how these specific concentrations were chosen for the study. Understanding the rationale behind the selection of these concentrations will provide insight into the experimental design and its relevance.

**Response:** Thanks for comment. We used the plant extract based on the doubled the fold of MTT value.

Comment #22: The authors should ensure that they cite relevant sources when discussing established methodologies used in the study. For instance, when referencing the MTT assay, Trypan Blue exclusion method, and DCFH-DA assay for ROS measurement, it is important to provide appropriate citations to support these methodologies. This will not only enhance the credibility of the manuscript but also guide readers to the original sources for further information on these techniques.

**Response:** Thank you for the feedback. We have added relevant references to support the methods used.

Comment #23: In line 130, the concentration unit used should be expressed in terms of per volume rather than just the weight of the substance. This presentation is important for clarity and accuracy in conveying the concentrations used in the study. I recommend revising this line to include the appropriate volume measurement alongside the weight.

**Response:** Thank you for pointing out the error. In our laboratory, we keep uisng the μg/mL. We will incorporate this suggestion in future study.

Comment #24: In line 139, the unit for the amount of cells used per well should be expressed more appropriately. Clear and consistent units are essential for ensuring accurate interpretation of the data. I recommend revising this line to specify the unit used for cell quantity, such as cells/well, to enhance clarity.

**Response:** Thank you for the suggestion. We have incorporated the recommended unit.

Comment #25: In line 141, it would be beneficial to specify what the AO/EtBr dual staining solution is, including the components and their respective functions. Additionally, please provide the ratio used for the assay. This information will enhance the clarity and reproducibility of the methodology for readers.

**Response:** We have included the Acridine Orange/Ethidium Bromide (Ao/EtBr) solution ratio and highlighted the importance of dual staining in the assay.

Comment #26: In line 145, under the subsection 'Intercellular ROS measurement,' please provide the full name of ROS and DCFH-DA before introducing their abbreviations. I recommend providing a brief explanation of the DCFH-DA assay. This will help readers who may not be familiar with the method to understand its purpose and significance in the context of the study.

**Response:** Yes, we have included the full names of DCFH-DA (2',7'-Dichlorofluorescin diacetate) and ROS (Reactive Oxygen Species), along with key points about the DCFH-DA assay.

Comment #27: In lines146, and 153, the unit for the amount of cells used per well should be expressed more appropriately. Clear and consistent units are essential for ensuring accurate interpretation of the data. I recommend revising this line to specify the unit used for cell quantity, such as cells per/well, to enhance clarity.

**Response:** We have incorporated the appropriate unit of measurement for cell quantity as suggested.

Comment #28: In line 161, the amount of cells used per well for the nuclear fate analysis was not mentioned.

**Response:** We have included the volume of cells used in the experiment.

Comment #29: In line 159, under the subsection ‘Nuclear fate analysis using Hoechst-33342 staining “, I recommend including a brief explanation of how Hoechst-33342 is prepared for staining. Providing details on the preparation process will enhance the reproducibility of the experiment and ensure that readers understand the methodology employed.

**Response:** Thank you for the feedback. We have provided more detailed information about the Hoechst-33342 staining method.

Comment #30: In line 166, under the subsection ‘Cell viability analysis using Propidium iodide“, I recommend including a brief explanation of how propidium iodide is prepared for staining. Providing details on the preparation process will enhance the reproducibility of the experiment and ensure that readers understand the methodology employed.

**Response:** Thank you for the feedback. We have implemented your suggestion.

Comment #31: On lines 223 – 227, under the Statistical Analysis subsection, the authors state that ANOVA tests were utilized for statistical analysis; however, there is no mention of the post hoc tests employed. Inclusion of the specific post hoc tests used is important for a complete understanding of the data analysis and to support the interpretations made in the manuscript.

**Response:** Thank you for the comment. Currently, we are using ANOVA for result analysis in our laboratory. For future studies, we plan to incorporate post-hoc tests for more detailed comparisons.

Comment #32: I recommend placing all figure legends directly beneath their respective figures. This arrangement would make it easier for reviewers to reference and evaluate the figures in relation to their legends, thereby improving the overall clarity and accessibility of the manuscript.

**Response:** Thank you for the feedback. We have added figure legends to each corresponding figure.

Comment #33: The discussion section is currently too lengthy. Please consider condensing the explanations of the findings and avoid repeating detailed results from the results section. To strengthen the interpretations, please incorporate citations from relevant literature to support your claims. In addition, the authors need to highlight how the findings contribute to scientific knowledge or practical applications and suggest how they might impact future research or policy decisions.

**Response:** Thank you for the suggestion. Based on the feedback from all authors, we have decided to retain the current format of the discussion section.

Comment #34: The conclusion effectively summarizes the key findings of the study. It highlights the significant cytotoxic effects of Otostegia fruticosa extract on MDA-MB-231 cells and emphasizes the mechanisms through which apoptosis and reduced cell viability occur. The mention of specific cellular changes, gene expression alterations, and protein analysis adds depth and supports the conclusions drawn.

**Response:** Thanks for the feedback. The suggestion has been carried out.

**Response to Reviewer #2**

***The constructive comments by the reviewer is really appreciated and it helped a lot to improve our manuscript. We have now revised the manuscript according to reviewer’s comment we have also answered each of your points below and hope the revised version might be suitable for the publication in this journal.***

Comment #1: The manuscript contains formatting and typographical errors. For example, the reference numbering in Line 59 appears incorrect, and the scientific name Otostegia fruticosa in Line 23 is not written consistently.

**Response:** Thank you for pointing out the error. It has been corrected.

Comment #2: The authors should provide complete information for all chemicals and reagents used, including the company name, city, and country.

**Response:** Thank you for pointing out the error. It has been corrected

Comment #3: Include the voucher specimen number and herbarium details to validate plant identification and authentication.

**Response:** We have provided more detailed information about the herbarium preparation.

Comment #4: Each protocol mentioned in the Materials and Methods section should be supported with appropriate references

**Response:** Thank you for the feedback. We have included the relevant references.

Comment #5: Details of all instruments used should be included (brand, model, city, and country). For instance, in the statement “The resulting extract was lyophilized and stored at -80°C,” the lyophilizer's brand and origin should be stated.

**Response:** Thank you for the feedback. We have included the necessary details.

Comment #6: The extraction procedure lacks critical details. Please specify the duration of extraction, solvent-to-material ratio, temperature, and any other relevant conditions.

**Response:** Thank you for the feedback. We have added the required information.

Comment #7: Concentrations used in assays are inconsistently reported. For example, the MTT assay uses a 24-hour incubation period, whereas other assays extend to 48 hours. Provide justification for the varying incubation periods and clearly explain the rationale for selecting specific concentrations. Additionally, report concentrations in µg/mL rather than as absolute amounts.

**Response:** Thanks for the comment. IC_50_ value is used to treat the cells by 24 hours. additionly, we want to see the effect of IC_50_ in prolonged manner. That why, we used 48 hours treatment by the same IC_50_ value. Thanks for understanding. And all authour suggested to display the current uint of concentration (µg/mL).

Comment #8: In Line 121, ensure that the percentage symbol (%) is included appropriately in the formula.

**Response:** Thank you. We have included the appropriate symbol.

Comment #9: Clarify the functional difference between the MTT assay and the trypan blue exclusion assay, particularly in the context of this study. Why did both assays need to be used in this study?

**Response:** Currently, scientists often use the MTT assay to determine IC_50_ values, while Trypan blue assay is employed to detect and quantify apoptotic cells through microscopy examination. Hence we adopted the Trypan Blue assay for detection of apoptotic cells. Thanks for understanding.

Comment #10: The use of different concentrations across experiments, such as the IC50 value in the intracellular ROS assay, needs to be justified.

**Response:** Agreed and justified.

Comment #11: Detail how the number of migrated and invaded cells was quantified in the respective assays.

**Response:** Thank you for the comment. The counting method has been added to the manuscript.

Comment #12: In Line 223, clarify the statistical approach used for multiple comparisons. Was a post hoc test performed?

**Response:** We performed ANOVA to clarify multiple comparisons.

Comment #13: In Line 208, specify the dilution ratio or concentration of the antibody used.

**Response:** We have specified the concentration of the antibody used.

Comment #14: Line 244: The conclusion that the extract causes cytoplasmic damage needs stronger evidence. The IC50 value is not clearly visible in the graph. Consider presenting the data using a dot plot instead of a bar graph for better clarity.

**Response:** Thank you for the comment. We have included red colour box for IC_50_ value highlight in the graph instead of modifying the bar graph.

Comment #15: Line 264: Apoptotic morphological changes should be clearly shown and labeled in the corresponding figure.

**Response:** Yes, we agreed and improved.

Comment #16: The manuscript would benefit from a discussion on the potential bioactive compounds present in the extract. Highlight known phytochemicals with anticancer properties and relate them to the observed effects, including apoptosis induction and suppression of metastasis-related proteins.

**Response:** Thanks for the comment. We've included the information.

We are grateful for giving us the opportunity to strengthen our manuscript with all your valuable comments and queries. We have worked hard to revise the manuscript and hope that the present revision persuades you to accept our submission. We look forward to hearing from you in due course regarding our submission and to respond any further questions and comments you may have.

*****
