# Supplementary material for: Ethanolic extract of Otostegia fruticosa induces ROS-dependent apoptosis and reduces migration of MDA-MB-231 cells in vitro
Source: PLoS One. 2026 May 26;21(5):e0349314. doi: 10.1371/journal.pone.0349314 (PMC13210379; doi:10.1371/journal.pone.0349314)
Supplement: Attachment — Submitted filename: renamed_216c8.docx [file pone.0349314.s003.docx]

**Author’s Response to the Editors and referees Comments**

**PONE-D-25-10876R2**

**Molecular insights of apoptotic/metastasis effects of Otostegia fruticosa forssk on triple-negative breast cancer cell (MDA-MB-231)**

| **S. No** | **Reviewer # 3: Comments** | **Author’s Response** |
| --- | --- | --- |
| 1 | "The manuscript titled Molecular insights of apoptotic/metastasis effects of Otostegia fruticosa forssk on triplenegative breast cancer cell (MDA-MB-231) ' adheres to scientific guidelines and demonstrates strong technical quality." | We sincerely thank the reviewer for the positive evaluation of our manuscript and for recognizing its scientific rigor and technical quality. We greatly appreciate your encouraging comments, which motivate us to further strengthen the clarity and impact of the study. |
| 2 | "Make sure to thoroughly review all tables for correct formatting and accurate data representation." | We thank the reviewer for this valuable suggestion. We have carefully re-examined all tables in the manuscript to ensure correct formatting, consistency, and accurate data representation. Necessary revisions have been made to improve clarity, alignment, and overall presentation quality. |
| 3 | "After reviewing all the revised comments from reviewers 1 & 2 to the list for accuracy and formatting, to strengthen the manuscript." | We sincerely thank the reviewer for this important suggestion. We have carefully re-evaluated all revisions made in response to the comments from Reviewers 1 and 2 to ensure accuracy, consistency, and proper formatting throughout the manuscript. Additional refinements have been implemented to further strengthen the clarity, coherence, and overall quality of the study. |
| 4 | A thorough grammatical review should be conducted, including checks for spacing, punctuation (e.g., commas), and spelling throughout the entire document." | We thank the reviewer for this valuable suggestion. The manuscript has undergone a thorough grammatical review to ensure accuracy and consistency in language usage. We have carefully corrected spacing, punctuation (including comma placement), spelling, and overall sentence structure throughout the entire document to improve clarity and readability. |
| 5 | "Overall, this is a well-structured and well-analyzed manuscript. The figures are clear, and future studies are recommended to further substantiate the findings." | We sincerely thank the reviewer for the positive and encouraging evaluation of our work. We greatly appreciate the recognition of the manuscript’s structure, analytical rigor, and figure clarity. We also agree that further studies will be valuable to expand and substantiate the present findings, and we have highlighted this perspective in the revised manuscript as part of the future research directions. |
| **S. No** | **Reviewer # 4: Comments** | **Author’s Response** |
| 1 | The abstract presents a clear overarching aim—to investigate the apoptotic and anti-metastatic properties of *Otostegia fruticosa* in triple-negative breast cancer (TNBC) cells (MDA-MB-231). The flow is generally logical, moving from objective → methods → key findings → conclusion. However, several sentences require refinement for clarity and scientific precision, and some terms are incorrectly used (e.g., “loss of nuclear fate”). Inclusion of minimal quantitative descriptors, and more cautious interpretation of mechanistic pathways are also vital.  • The abstract briefly mentions the key assays used (MTT, fluorescence staining, trypan blue exclusion, DCFH-DA staining, functional assays, qRT-PCR, immunofluorescence, Western blot), which is appropriate. However:  The description of experimental approaches is too broad and lacks specificity.  For example, “functional assays” should specify migration, wound healing, or transwell assays.  “Loss of nuclear fate” is not a standard apoptotic descriptor; it should be revised to “nuclear condensation and fragmentation.”  • Quantitative results (e.g., fold-changes, significance levels) are absent. Although an abstract need not be data-heavy, the absence of any quantitative markers weakens scientific rigor. | We sincerely thank the reviewer for the thorough and constructive evaluation.  Specifically, we have replaced the non-standard term “loss of nuclear fate” with the scientifically appropriate description “nuclear condensation and fragmentation.” The description of experimental procedures has been refined for greater specificity, with “functional assays” now clearly defined as wound healing and transwell migration assays.  Furthermore, all authour of this study agreed to mention the statistical significance at the figure legend.  These revisions collectively enhance the clarity, precision, and scientific quality of the abstract. |
| 2 | The abstract concludes that Otostegia fruticosa induces apoptosis and suppresses metastasis through modulation of AKT signaling and apoptosis-related genes. Strengths include:  • The mechanistic pathway is not sufficiently justified. If PI3K/Akt inhibition is highlighted, the abstract should mention whether phospho-Akt levels decreased.  • It is unclear whether Otostegia fruticosa was fractionated or characterized phytochemically; this limits reproducibility. | We sincerely thank the reviewer for this insightful and constructive comment. In response, we have revised the concluding statements in the abstract to ensure that the mechanistic claims are presented more cautiously and are directly supported by our experimental findings. “P-Akt level was downregulated”  Furthermore, to improve transparency and reproducibility, we have explicitly stated in the revised manuscript that an ethanolic extract of Otostegia fruticosa was used in this study. The extraction method has been clearly described in the Materials and Methods section to enhance methodological clarity.  These revisions strengthen the mechanistic justification and improve the reproducibility and scientific precision of the manuscript. |
| 3 | “Hyper ROS activity” should be phrased as “excessive ROS generation.” | We thank the reviewer for this valuable suggestion. The terminology has been corrected accordingly, and “hyper ROS activity” has been replaced with the more scientifically appropriate term “excessive ROS generation” throughout the manuscript to ensure clarity and accuracy. |
| 4 | No mention of statistical significance. | We thank the reviewer for highlighting this important point.  All authour of this study agreed to mention the statistical significance of results placed in the bottom of figure legend as laboratory pratices. Now, all figure legends contains the statistical significance. Thanks you. |
| 5 | Several sentences are long and contain redundancies. | We thank the reviewer for this helpful observation. The manuscript has been carefully revised to improve sentence structure and eliminate redundancies. Long and complex sentences have been restructured for greater clarity, conciseness, and readability, thereby enhancing the overall flow of the manuscript. |
| 6 | Otostegia fruticosa should be italicized as a scientific plant name. | We thank the reviewer for highlighting this oversight. The scientific name Otostegia fruticosa has been properly italicized throughout the manuscript in accordance with standard taxonomic conventions. |
|  | THE BODY OF THE MANUSCRIPT |  |
| 1 | Composition and standardization of the extract (fundamental).  • No phytochemical characterization (HPLC, LC-MS, GC-MS) or quantification of active component(s). Reporting activity of an undefined crude extract without standardization prevents reproducibility and interpretation  The Authors should Provide voucher specimen number, deposit info, and a full phytochemical profile. | We sincerely thank the reviewer for raising this important methodological concern. We fully agree that extract characterization and proper documentation are essential for reproducibility and scientific rigor.  In the revised manuscript, we have clearly specified that the study was conducted using an ethanolic crude extract of Otostegia fruticosa. The extraction procedure has been described in detail in the Materials and Methods section to improve methodological transparency.  Furthermore, we have added the herbarium voucher specimen number along with the deposition details to ensure botanical authentication and traceability of the plant material.  While a comprehensive phytochemical profiling (e.g., HPLC/LC-MS/GC-MS-based characterization and quantification of active constituents) was beyond the scope of the present study, we have now included a discussion of previously reported bioactive compounds from Otostegia fruticosa in the revised manuscript to provide phytochemical context. We also acknowledge this limitation explicitly and highlight the need for future studies focusing on extract standardization and compound-level characterization.  In our previous revision of this manuscript, we carefully addressed this technical point to reviewer 1 and 2.  These additions improve reproducibility, transparency, and scientific interpretation of the findings. |
| 2 | Controls and vehicle information are missing or unclear.  • The methods do not state the extract solvent (final vehicle concentration), whether vehicle controls (e.g., ethanol or DMSO) were included, or whether solvent affected cell viability. Without vehicle controls, cytotoxicity attribution to the extract is unreliable.  Authors should State solvent, final % v/v, and include vehicle controls in all assays. | We sincerely thank the reviewer for highlighting this important methodological point. In the revised manuscript, we have clarified the extraction and vehicle preparation procedures to ensure transparency and reproducibility.  Briefly, ethanol was used for the initial extraction process; however, the solvent was completely removed using lyophilization. The dried extract was subsequently reconstituted in cell culture–grade sterile water prior to use in all in vitro experiments. Therefore, no residual ethanol was present during treatment, and no organic solvent vehicle (e.g., DMSO or ethanol) was introduced into the cell culture system.  We have explicitly stated this in the Materials and Methods section. Additionally, control groups treated with an equivalent volume of sterile cell culture-grade water were included in all assays to ensure that observed effects were attributable solely to the plant extract.  These clarifications have been incorporated to strengthen methodological transparency and ensure accurate interpretation of cytotoxicity and mechanistic findings. Thanks for understanding. |
| 3 | Insufficient description and no-mention of biological replicates and statistics.  • Manuscript states “mean ± SD” but does not specify n (technical vs biological replicates; how many independent experiments). Authors should state Which statistical tests were used for each figure? Were assumptions (normality, equal variance) checked? Provide exact p-values, sample sizes (n =?). | We sincerely thank the reviewer for highlighting the need for greater clarity regarding statistical analysis and experimental replication. We fully agree that transparent reporting of sample size and statistical methodology is essential for scientific rigor.  In the revised manuscript, we have clearly specified the number of biological replicates (independent experiments) and technical replicates performed for each assay. The sample size (n) is now explicitly indicated in the figure legends and/or Methods section, as appropriate.  Furthermore, we have detailed the statistical tests used for each analysis (e.g., one-way or two-way ANOVA followed by appropriate post hoc testing) in the Statistical Analysis subsection. Where applicable, we have clarified that assumptions of normality and homogeneity of variance were evaluated prior to applying parametric tests.  These revisions substantially improve the transparency, reproducibility, and statistical robustness of the study. |
| 4 | Overreliance on single-method, low-specificity assays for apoptosis  • MTT, AO/EtBr, and trypan blue are supportive but insufficient to claim apoptosis vs necrosis. No flow cytometry (Annexin V/PI) or caspase activity assay (colorimetric/fluorometric) is shown.  • If Authors had performed Annexin V/PI assay by flow, caspase-3/-7 activity, they should be included but if such assay was not performed, then their language should be mild or specifically on cell viability and death instead of apoptosis. | We sincerely thank the reviewer for this important and insightful comment. We acknowledge that assays such as MTT, AO/EtBr staining, and trypan blue exclusion provide supportive but not definitive evidence of apoptosis, and that more specific assays (e.g., Annexin V/PI flow cytometry or caspase activity assays) would further strengthen mechanistic validation.  As these additional assays were beyond the scope of the present study, we have carefully revised the manuscript to moderate our conclusions. Specifically, we have adjusted the language to avoid overinterpretation and now describe the findings more cautiously in terms of reduced cell viability and induction of apoptotic-like morphological changes, rather than definitively claiming apoptosis where not directly confirmed by gold-standard assays.  These revisions ensure that the claims remain evidence-based and scientifically balanced. |
| 5 | Mechanistic claims (PI3K/Akt, PTEN) are correlative.  • The manuscript infers pathway inhibition from decreased mRNA and p-Akt Western blot. This correlation is insufficient to conclude causal involvement. The pathway is only suspected except a PTEN knockdown/overexpression, or rescue experiments (constitutively active Akt) had been performed to validate that apoptosis/migration effects are mediated via PI3K/Akt. | We sincerely thank the reviewer for this insightful and scientifically rigorous comment. We fully acknowledge that the current data demonstrate an association between extract treatment and modulation of PI3K/Akt pathway components (including altered mRNA expression and reduced phospho-Akt levels), but do not establish direct causal involvement.  In response, we have carefully revised the manuscript to moderate the mechanistic interpretation. The language has been adjusted to reflect that our findings suggest or are consistent with modulation of the PI3K/Akt signaling pathway, rather than definitively proving pathway-dependent causality.  These revisions ensure that our conclusions remain evidence-based, cautious, and scientifically appropriate. |
| 6 | Western blot reporting is inadequate.  o No full uncropped blots are shown. Actin B is claimed as loading control but were the blots normalized? Authors should describe antibodies, dilutions, exposure times. Provide uncropped images in Supplementary. Include biological replicates (n≥3) and statistical analysis of band quantification. | We sincerely thank the reviewer for this important comment regarding Western blot reporting and transparency.  In the revised manuscript, we have substantially improved the methodological description and data presentation.  Specifically:  Detailed information regarding primary and secondary antibodies (including source, catalog numbers, and dilutions) has been added to the Materials and Methods section (Chemical used).  Exposure conditions and detection methods have been clearly described experimental section.  β-Actin was used as a loading control, and densitometric analysis was performed with normalization of target protein bands to β-Actin.  Nullify method is,   1. Control Target band intensity/ control beta actin intensity. 2. Treated Target band intensity/ Target beta actin intensity. 3. Target value/ Control value= fold level value 4. Then, Mean, SD, N were used generated Graphs.   Raw western blot images have been provided in the Supplementary Information to ensure transparency and data integrity (Specific region of interest of blot membrane was cutted and incubated with specific primary antibody separatively. Those cutted membrane wasused to develop band).  These revisions enhance reproducibility, transparency, and methodological rigor of the Western blot experiments. |
| 7 | qRT-PCR methodology  • No information on primer efficiencies, or whether assays were run in technical triplicate. Some primer sequences appear malformed (e.g., BCL2 forward contains an extra ‘F-’). Validation of primers can be included. | Thank you for pointing out the error. We have corrected it. |
| 8 | Choice of HEK-293 as “normal” control is problematic.  • HEK-293 are embryonic kidney cells (transformed) and are not an appropriate non-tumour breast control. If aiming to demonstrate cancer selectivity, use non-transformed human mammary epithelial cells (HMEC) or MCF-10A. If HEK-293 are retained, justify explicitly. | We sincerely thank the reviewer for this important and thoughtful comment regarding the selection of control cell lines.  We acknowledge that HEK-293 cells are immortalized embryonic kidney cells and do not represent normal mammary epithelial cells. In the present study, HEK-293 cells were included to provide a general non-cancerous human cell comparison for preliminary toxicity evaluation of the Otostegia fruticosa extract, rather than to serve as a tissue-matched normal breast epithelial control.  We agree that non-transformed mammary epithelial cell lines such as HMEC or MCF-10A would provide a more physiologically relevant comparison for assessing cancer selectivity in breast cancer models. This limitation has now been explicitly acknowledged in the revised result section. We have clarified that the use of HEK-293 cells was intended to assess general cytotoxic selectivity and not tissue-specific normal breast comparison.  Additionally, HEK-293T cells were not used due to their SV40 large T-antigen expression and higher tumorigenic potential, which would not be suitable for evaluating non-cancerous cell toxicity. |
| 9 | Invasion/migration assays lack critical controls and quantification details.  • Matrigel concentration and coating method are only briefly stated. Were differences in proliferation accounted for (reduced proliferation could mimic reduced migration)?  Wound-healing assays must be performed in serum-free or mitomycin-C-treated conditions to prevent proliferation confounders. Authors should provide quantitative metrics (% wound closure), replicate numbers, and images with scale bars. | We sincerely thank the reviewer for this important and constructive comment regarding the migration and invasion assays.  In the revised manuscript, we have provided a detailed description of the Matrigel concentration, coating procedure, and incubation conditions used for the transwell invasion assays in the Materials and Methods section to improve reproducibility.  To address the potential confounding effect of proliferation, we have clarified the experimental conditions under which the migration assays were performed (including serum-free conditions). This information has now been explicitly stated in the Methods section to ensure accurate interpretation of migration-specific effects.  Quantification data for wound healing assay is already prresented.  Furthermore, we have included quantitative analysis of invasion results (invasion percentage), along with the number of biological replicates performed (n = 3). Representative images now include appropriate scale bars, and statistical analysis of the quantified data has been added in figure section.  These revisions significantly improve methodological transparency, quantitative rigor, and confidence in the interpretation of the migration and invasion findings. |
| 10 | Claims exceed data (language and novelty).  • The manuscript repeatedly generalizes to “mechanism” and therapeutic potential without isolation of active compounds or in vivo validation. Authors should rephrase claims to be proportional to the in vitro evidence instead of generalising.  Finally, authors should limit redundant statements: The Discussion restates results extensively — tighten and focus on interpretation, limitations, and next steps  NOTE: if ROS dependency is shown, then the title may be rephrased to:  Ethanolic extract of Otostegia fruticosa induces ROS-dependent apoptosis and reduces migration of MDA-MB-231 cells in vitro” but only use if ROS dependency is shown | We sincerely thank the reviewer for this thoughtful and constructive comment regarding the scope and framing of our conclusions.  In the revised manuscript, we have carefully moderated the language throughout the Abstract, Results, and Discussion sections to ensure that our claims remain proportional to the in vitro nature of the study. Specifically, references to definitive “mechanisms” and therapeutic implications have been revised to more cautious phrasing (e.g., “suggests involvement,” “is associated with,” or “demonstrates potential in vitro activity”), thereby avoiding overgeneralization beyond the presented data.  Regarding the title, we carefully considered the reviewer’s suggestion. The title has been revised (as appropriate based on the experimental evidence) to better reflect the in vitro scope and the role of ROS, while ensuring that claims remain evidence-based. |
| **S. No** | **Reviewer # 5: Comments** | **Author’s Response** |
| 1 | Please revise the abstract to remove the section labels “Background:,” “Aim:,” “Method and Results:,” and “Conclusion:.” The abstract should be presented as a single, cohesive paragraph without these headings. | We sincerely thank the reviewer for this helpful suggestion. In accordance with the journal’s formatting guidelines, we have removed all section labels (e.g., “Background,” “Aim,” “Methods and Results,” and “Conclusion”) and revised the abstract into a single, cohesive paragraph. The revised abstract now maintains logical flow and clarity while adhering to the required structural format. |
